# Phenotypic and Allelic Frequencies of ABO and Rh(D) Blood Antigens in Ghana: A Systematic Review

**DOI:** 10.1002/iid3.70112

**Published:** 2024-12-25

**Authors:** Charles Nkansah, Felix Osei‐Boakye, Samuel K. Appiah, Gabriel Abbam, Moses Banyeh, Samira Daud, Richard V. Duneeh, Simon B. Bani, Boniface N. Ukwah, Charles A. Derigubah, Victor U. Usanga, Emmanuel Appiah‐Kubi, Ejike F. Chukwurah

**Affiliations:** ^1^ Department of Haematology School of Allied Health Sciences University for Development Studies Tamale Ghana; ^2^ Department of Medical Laboratory Science Faculty of Health Sciences and Technology Ebonyi State University Abakaliki Nigeria; ^3^ Department of Medical Laboratory Technology Faculty of Applied Science and Technology Sunyani Technical University Sunyani Ghana; ^4^ Department of Biomedical Laboratory Sciences School of Allied Health Sciences University for Development Studies Tamale Ghana; ^5^ Department of Medical Laboratory Science School of Allied Health Sciences University of Health and Allied Sciences Ho Ghana; ^6^ Department of Medical Laboratory Technology School of Applied Science and Arts Bolgatanga Technical University Bolgatanga Ghana

**Keywords:** ABO blood group, allelic frequency, Ghanaian population, Rh(D) blood group

## Abstract

**Background:**

ABO and Rh blood group systems are the most significant blood group systems recognized by the International Society of Blood Transfusion and are widely used for clinical and anthropological purposes. This systematic review determined the distribution and allelic frequency of ABO and Rh(D) antigens in Ghana.

**Methods:**

Literature searches were performed in PubMed, Google Scholar, Web of Science, and ScienceDirect, up to February 20, 2024, and included studies published from 2000 to 2024 in all regions of Ghana. The search terms used to retrieve the preferred literature were “Blood Group/Antigen” and “ABO and Rh(D)” and “Distribution/Frequency/Prevalence,” coupled with the names of the different regions/districts/municipalities in Ghana. Similar blood group individuals from all the regions were added, and countrywide data were gathered. The Hardy−Weinberg model was used to estimate the allelic frequency of blood antigens.

**Results:**

Blood group O (54.72%) was the predominant group in the Ghanaian population, followed by B (21.74%), A (19.65%), and AB (3.89%). Rh(D) antigen was present in 92.28% of the population, and only 7.72% were Rh(D) negative. The calculated allelic frequencies of A, B, O, Rh(D) positive, and Rh(D) negative were 0.1227, 0.1376, 0.7397, 0.7222, and 0.2778 for I^A^(p), I^B^(q), i(r), I^D^(v), and I^d^(u), respectively.

**Conclusion:**

The phenotypic frequency of the ABO blood group occurred in the pattern O>B>A>AB, and the prevalence of the Rh(D) negative blood group was 7.72% in Ghana. Future nationwide studies are recommended to assess the distribution of ABO, Rh, and other blood group systems.

## Graphical abstract

Blood group O is predominant in the Ghanaian population, and the blood antigens occur in the pattern O>B>A>AB. Prevalence of Rh(D)‐negative is 7.72%. Phenotypic frequencies of blood antigens A and B vary in different regions of Ghana.

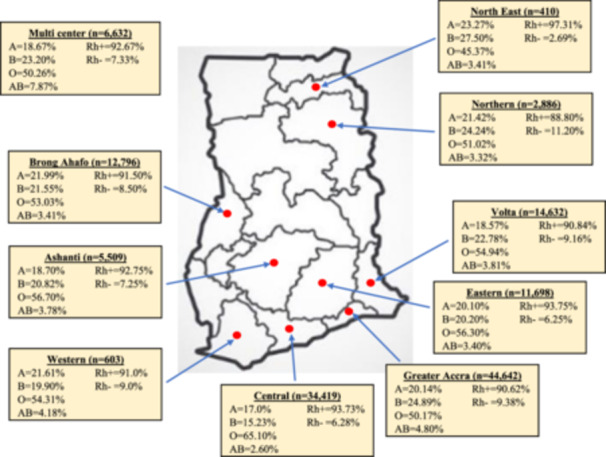

## 1 Introduction

The discovery of the ABO blood group system in the early 20th century by Karl Landsteiner brought great relief in clinical practice, as testing of donor blood before transfusion significantly reduced complications related to blood transfusion reactions and enhanced the safety of blood transfusion medicine. Karl Landsteiner identified three blood group antigens, A, B, and O (formerly named C), and in 1902, two students of Karl Landsteiner, De Castello and Sturli, identified the blood group AB [1, 2]. The gene that codes for ABO blood groups is situated on a single locus of the long arm of chromosome 9 (9q34), and its inheritance follows the Mendelian pattern [1]. Three broad alleles of the ABO gene have been identified: *I* ^*A*^ expresses A antigen (containing α‐1,3‐*N*‐acetylgalactosamine), *I* ^*B*^ expresses B antigen (containing α‐1,3‐galactose), and *i* expresses no ABO antigen and hence described as O (expresses only H‐antigen precursor), where *I* represents isoagglutinogen. Antigens A and B require the presence of a preformed H antigen which serves as a substrate for ABO glycosyltransferases [3]. The dominance of both *I* ^*A*^ and *I* ^*B*^ over *i* has been recognized, with only homozygous *ii* individuals belonging to blood group O and people with *I* ^*A*^ *I* ^*B*^ having both phenotypes because of their codominance have the blood group AB [4]. Thus, ABO antigens are carbohydrate molecules on the surfaces of erythrocytes and other cells such as platelets, neurons, vascular epithelium, and endothelium [5].

The Rh blood group system remains the second most important blood group, after the ABO system in blood transfusion medicine, and was discovered by Karl Landsteiner and Wiener in 1939−1940. Of the over 50 Rh antigens, Rh D and Rh CE are the most medically significant, and their expression is controlled by closely linked genes on the short arm of chromosome 1 [3]. A total of 43 blood group systems and 345 red cell antigens have been recognized by the International Society of Blood Transfusion (ISBT), with nine of them—ABO, Rh, Kell, Duffy, Kidd, MNS, P, Lewis, and Lutheran—capable of inducing hemolytic disease of the fetus and newborns and hemolytic transfusion reactions. Although ABO, Rh, and Kell are mostly immunogenic compared to others, ABO and Rh are the most significant blood antigens in blood transfusion services [6]. These blood antigens are inherited via Mendelian inheritance, found primarily on red cell surfaces, and represent biological traits that remain unchanged for life in healthy individuals [1]. The knowledge about ABO and Rh blood group systems are not only beneficial in clinical transfusion practices, but also essential for organ transplantation, population genetics studies, forensic investigations, paternity identification, population migration processes, gene flow, genetic hitchhiking, and anthropological studies [7]. The distribution of ABO and Rh blood antigens could be influenced by genetic drift, geographical and environmental factors, natural selection for population survival, ethnicity, and race [7, 8]. The pathogenesis of infectious diseases, malignancies, and metabolic disorders has been associated with ABO and Rh blood groups. Rare alterations in ABO and Rh blood groups of individuals with certain diseases have been reported [9, 10, 11].

Ghana is partitioned into 16 administrative regions and has approximately 33,107,275 population. Majority (44%) of the Ghanaian population belong to Akan race, with Mole‐Dagbon, Ewe, Ga‐Adangbe, and others being 18.5%, 13.9%, 7.1%, and 16.5%, respectively. Also, the population of Ghana has a great religious diversity such as 71.3% Christians, 19.9% Muslims, 3.2% Traditionalists, and 5.6% others [12]. The various population groups may be similar or vary with regard to genetic traits, which affects the distribution of blood antigens [3]. Variable frequencies of ABO and Rh antigens have been reported among populations in different regions of Ghana [13, 14, 15, 16, 17, 18, 19, 20]. Although some studies have reported the distribution of ABO and Rh(D) in specific regions of Ghana, there is still no available study that focused on larger populations to reflect a national picture. Three multicenter studies [19, 21, 22] included small areas in two to four regions at a time and involved 6332 participants; however, this sample size is small for generalizability. Again, so far, there are no relevant data in the Ghanaian population regarding blood antigen distribution and their corresponding allelic frequencies.

Therefore, this systematic review explored the phenotypic and allelic frequencies of ABO and Rh(D) blood antigens in Ghana by analyzing various studies on the distribution of blood antigens in different regions of Ghana, over a period of 24 years, from January 2000 to February 2024. These findings will provide national and regional blood bank information and improve the safety of blood transfusion services in Ghana.

## 2 Methods

This systematic review adhered to the guidelines enshrined in the Preferred Reporting Items for Systematic Reviews and Meta‐Analysis (PRISMA) statement [23]. The search aimed to retrieve studies that reported frequency or distribution of ABO and Rh(D) blood antigens in Ghana.

### 2.1 Database Searches/Literature Search

In this systematic review, literature searches were performed using various search engines, including PubMed, Google Scholar, Web of Science, and ScienceDirect, from January 1, 2024, to February 20, 2024. The search terms used to retrieve the literature were “Blood Group/Antigen” and “ABO and Rh(D)” and “Distribution/Frequency/Prevalence” coupled with the names of the different regions/districts/municipalities in Ghana. The literature search was limited to studies published from 2000 to 2024. The preliminary literature search was attempted by C.N., but all the searched literatures were screened and reviewed autonomously by C.N. and F.O.‐B. for probable eligibility.

### 2.2 Criteria for Eligibility of Studies

This systematic review included studies that assessed the distribution, frequency, or prevalence of ABO and Rh(D) blood group antigens among Ghanaians from January 1, 2000, to February 20, 2024. Animal studies, studies that sampled cord blood for the determination of ABO and Rh(D) blood groups, and studies that assessed non‐ABO and Rh(D) blood groups were excluded from this review.

### 2.3 Data Extraction and Grouping

The selected studies were exported to Mendeley version 1.17.11 for further screening and analysis. The selection of the final studies used in the review was systematically performed by following this pattern: duplicate articles were deleted, titles and abstracts of the residual studies were screened, nonessential titles and abstracts were removed, residual studies were assessed to obtain complete text, and studies with complete data were selected. Relevant data were extracted from the selected studies stored in Mendeley using standardized SPSS spreadsheet. The extracted data included names of authors, publication year, region of the study, district/municipality or hospital where the study was conducted, study design, sample size, participant type, method of blood typing, and ABO and Rh(D) frequencies observed. The studies were grouped based on 16 regions of Ghana [12]. All studies published on the distribution/prevalence/frequency of ABO and Rh(D) blood antigens from one region were assembled and organized in a common table. Individuals with matching blood antigens from all studies published in a particular region were added to assess the frequency of blood antigens of that region. Furthermore, the national distribution of ABO and Rh(D) was evaluated based on the number of individuals with identical blood antigens from all regions. Finally, the percentage frequency/distribution of blood antigens was estimated from the total number of participants.

### 2.4 Determination of Allelic Frequency

The Hardy−Weinberg model of quantitative genetics was used to estimate allelic frequencies of various blood antigens [3, 7]. This principle further predicted the maximum probability ratio with the hypothesis that three alleles of a distinct gene (A, B, and O) determined the ABO blood group system, with A and B being autosomal‐dominant over the O gene, and co‐dominant with each other. The expected phenotypic frequencies of various blood antigens were calculated using the allelic frequency outcomes. The difference between the observed and allelic frequencies of the blood antigens was assessed using the chi‐square test.

### 2.5 Quality Assessment of the Selected Studies

The critical appraisal tools described by the Joana Briggs Institute (JBI) were used to assess the quality of the included studies. The JBI tool containing the checklists for critical appraisal had 22 study design–based items, including title/abstract, background/rationale, specific objectives, study designs, study setting, participant, variables, data source management, study size, quantitative variables, statistical methods, participants selection, descriptive data, outcome data, main results, other analysis, key findings, limitation(s), interpretation, generalizability, and source of funding for the study. Individual studies were assessed for quality based on the 22 items listed above and were scored by dividing each study score by the total score (22) and expressed as a percentage. The quality of a study was described as low, moderate, or high when its final percentage scores were < 50%, 50%−75%, and > 75%, respectively.

## 3 Results

### 3.1 Search Results

**Figure 1:**
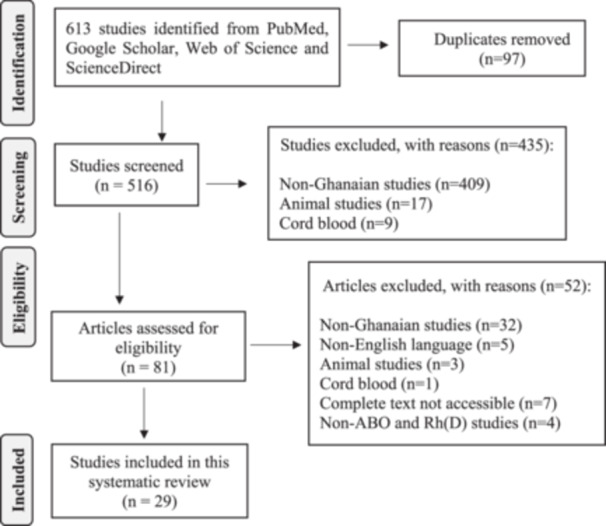
Flowchart indicating the studies screened, selected, and included in accordance with PRISMA.

In total, 613 studies were identified from PubMed, Google Scholar, Web of Science, and ScienceDirect. The titles and abstracts of 516 studies were screened after 97 duplicates were deleted. After screening titles and abstracts of the 516 studies, 435 studies were deemed ineligible and were excluded, and the remaining 81 studies were subjected to final full‐text eligibility assessment. After assessing the full‐text articles, another 52 studies were excluded. Finally, 29 full‐text studies were considered eligible for this systematic review. The 29 studies included were published in English language from 11 out of the 16 administrative regions of Ghana, from January 1, 2000, to February 20, 2024. During the inclusion period from 2000 to 2024, the distribution of ABO and Rh had not been reported in five regions (Upper East, Savanna, Ahafo, Bono East, and Western North regions) of Ghana (Figure 1).

### 3.2 Characteristics of the Studies Included in the Systematic Review

The total sample size of the 29 studies included in this systematic review was 134,227, with 62,150 (46.3%) males and 36,657 (27.3%) females; however, four studies with a total sample size of 35,420 (26.4%) did not specify the numbers for both sexes. Most of the studies recruited adults only (19/65.6%) [13, 14, 16, 19, 20, 22, 23, 24, 25, 26, 27, 28, 29, 30, 31, 32, 33, 34, 35] and were published in the last decade (26/89.7%) [10, 14, 15, 16, 17, 18, 19, 20, 22, 24, 25, 26, 27, 28, 29, 31, 32, 33, 34, 35, 36, 37, 38, 39, 40]. Twelve (41.4%) and 11 (37.9%) of the included studies were conducted in the middle belt [13, 15, 16, 18, 24, 27, 30, 32, 33, 34, 35, 40] and southern parts [17, 20, 25, 26, 28, 36, 37, 38, 39, 41] of Ghana, respectively, while three (10.3%) were multicenter studies [19, 21, 22].

Although almost half of the studies did not indicate methods for blood typing, eight (27.6%) and six (20.7%) of the studies confirmed blood antigens using the tube [10, 22, 25, 29, 32, 34, 38, 41] and tile [17, 21, 26, 27, 31, 36] methods, respectively, whereas one study performed the procedure through molecular techniques [15]. Based on the JBI quality assessment tools, 23/29 (79.3%) [10, 13, 14, 15, 16, 18, 19, 22, 24, 26, 27, 28, 29, 31, 33, 34, 35, 36, 37, 38, 39] of the included studies were of high quality, while six (20.7%) [17, 20, 25, 30, 32, 41] were of moderate quality. None of the selected studies was of low quality; hence, all studies were included in the systematic review (Table 1).

**Table 1:** Characteristics of the studies included in the systematic review.

Variables	Categories	Frequency	Percentage (%)
Sex	Females	36,657	27.3
	Males	62,150	46.3
	Not stated	35,420	26.4
Geographical location	Southern	11	37.9
	Middle belt	12	41.4
	Northern	3	10.3
	Multicenter	3	10.3
Type of participants	Children	3	10.3
	Adults	19	65.5
	Pregnant women	2	6.9
	All ages	5	17.2
Year of publication	2000−2013	3	10.3
	2014−2024	26	89.7
Method of determining blood group	Tile	6	20.7
	Tube	8	27.6
	Molecular	1	3.4
	Not stated	14	48.3
Quality of the included studies	High	23	79.3
	Moderate	6	20.7
	Low	0	0

### 3.3 National Distribution of ABO and Rh(D) Blood Groups in Ghana

**Figure 2:**
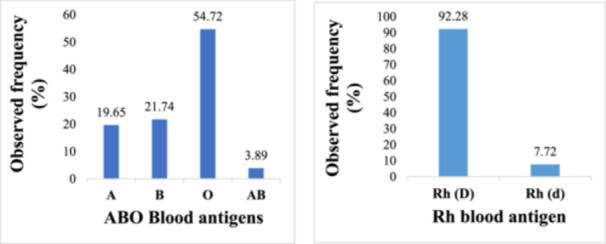
National distribution of ABO and Rh(D) blood groups in Ghana.

Of the 29 studies in Ghana included in this review, a total of 134,227 individuals were used to estimate the blood antigen distribution and allelic frequencies of every blood antigen in Ghana. The overall distribution of ABO blood antigens in Ghana was as follows: O (54.72%), B (21.74%), A (19.65%), and AB (3.89%), in the order O>B >A>AB. Regarding the Rh blood group system, 92.28% of individuals had the Rh(D) antigen, with only 7.72% being Rh(D) negative (Figure 2).

### 3.4 Region‐Specific Observed Frequencies of ABO and Rh(D) Blood Antigens in Ghana

**Figure 3:**
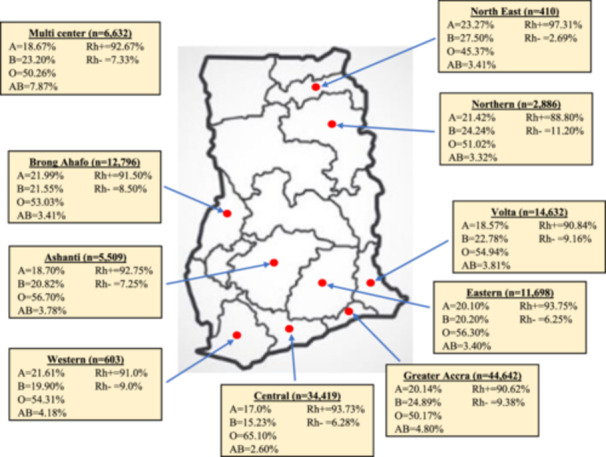
A map indicating the distribution (%) of ABO and Rh(D) antigens in different regions of Ghana.

The maximum number of studies used in this review were published in Greater Accra [17, 25, 38, 39, 41] and Ashanti [13, 15, 24, 32, 35] regions, but the Greater Accra region had the highest number of populations (44,642) covered in the studies. The distribution of blood antigens was calculated for nine regions, in addition to three multicenter studies in Ghana. Blood group O was predominant in all regions involved in this review. Blood antigen B was the second most common blood antigen in the Greater Accra (24.89%), Volta (22.78%), Ashanti (20.82%), Northern (24.24%), and North East (27.56%) regions and two inter‐regional studies (Ashanti; Upper West and Eastern [28.01%]; and Upper West, Greater Accra, Oti, and Northern [23.48%]). On the other hand, blood group A was second to O in the Central (17.0%) and Western (21.61%) regions and in one multicenter study (Ga East and Central [20.10%]). The distribution of blood antigens A and B was similar in the Eastern (A: 20.10% vs. B: 20.20%) and Brong Ahafo (A: 21.99% vs. B: 21.55%) regions. Individuals with the Rh(D) antigen were more prevalent in the North East (97.31%), Eastern (93.75%), and Central (93.73%) than in other regions of Ghana (Table 2). A map indicating the distribution (%) of the ABO and Rh(D) antigens in different regions of Ghana is presented in Figure 3.

**Table 2:** Region‐specific observed frequencies of ABO and Rh(D) blood antigens in Ghana.

Regions	Districts/area	A	B	O	AB	Comments	Rh+	Rh−	Total	References
Greater Accra	KBTH	21.27	22.05	54.60	2.08	O>B>A>AB	94.06	5.94	1533	[25]
	Accra	20.70	24.30	50.0	5.0	O>B>A>AB	93.79	6.21	42,317	[38]
	KBTH	19.80	26.40	49.60	4.20	O>B>A>AB	—	—	121	[41]
	Shai Osudoku	17.90	27.70	46.70	7.6	O>B>A>AB	—	—	463	[39]
	Adenta	21.0	24.0	50.0	5.0	O>B>A>AB	84.0	16.0	208	[17]
	Total	20.14	24.89	50.17	4.80	O>B>A>AB	90.62	9.38	44,642	
Central	UCC	21.40	17.10	58.10	3.40	O>A>B>AB	90.60	9.40	117	[26]
	Winneba	23.50	17.50	56.0	3.0	O>A>B>AB	92.20	7.80	166	[30]
	Cape Coast	11.30	12.90	75.0	0.80	O>B>A>AB	96.30	3.70	240	[36]
	Cape Coast	11.80	13.40	71.30	3.50	O>B>A>AB	95.80	4.20	33,896	[20]
	Total	17.00	15.23	65.10	2.60	O>A>B>AB	93.73	6.28	34,419	
Volta	HoTH	16.54	19.85	59.19	4.41	O>B>A>AB	88.97	11.03	272	[28]
	Volta	20.60	25.70	50.70	3.20	O>B>A>AB	92.70	7.30	14,360	[37]
	Total	18.57	22.78	54.94	3.81	O>B>A>AB	90.84	9.16	14,632	
Western	Western	18.93	23.21	50.71	7.14	O>B>A>AB	—	—	280	[27]
	West Akim	24.10	16.40	58.20	1.20	O>A>B>AB	91.0	9.0	323	[33]
	Total	21.61	19.90	54.31	4.18	O>A>B>AB	91.0	9.0	603	
Eastern	Birim North	21.30	20.80	54.30	3.80	O>A>B>AB	95.0	5.0	400	[30]
	Eastern	18.90	19.60	58.30	3.0	O>B>A>AB	92.50	7.50	11,298	[16]
	Total	20.10	20.20	56.30	3.40	O>B=A>AB	93.75	6.25	11,698	
Ashanti	Kumasi	22.70	21.60	53.10	2.70	O>A>B>AB	89.20	10.80	515	[24]
	Asante Akim North	18.0	22.60	55.40	4.0	O>B>A>AB	—	—	839	[15]
	Kumasi	20.82	18.52	57.44	3.20	O>A>B>AB	97.70	2.30	437	32
	Offinso north	12.30	20.40	66.40	0.90	O>B>A>AB	93.80	6.20	3306	[13]
	KNUST Hospital	19.70	26.0	51.20	3.10	O>B>A>AB	90.30	9.70	412	[35]
	Total	18.70	20.82	56.70	3.78	O>B>A>AB	92.75	7.25	5509	
Brong Ahafo	Sunyani	13.30	16.40	69.80	0.50	O>B>A>AB	89.90	10.10	6847	[40]
	Sunyani	23.0	24.90	48.10	4.0	O>B>A>AB	93.10	6.90	5089	[34]
	Wenchi and Tain	29.60	23.30	41.10	5.70	O>A>B>AB	—	—	860	[18]
	Total	21.99	21.55	53.05	3.41	O>A=B>AB	91.5	8.50	12,796	
Northern	TTH	29.80	23.80	40.50	6.0	O>A>B>AB	—	—	84	[29]
	Kpandai	13.06	24.70	61.60	0.64	O>B>A>AB	88.80	11.20	2802	[14]
	Total	21.42	24.24	51.02	3.32	O>B>A>AB	88.80	11.20	2886	
North East	West Mamprusi	23.27	27.56	45.37	3.41	O>B>A>AB	97.31	2.69	410	[10]
Multicenter	Kumasi, Wa, and Koforidua	17.59	28.01	49.19	5.21	O>B>A>AB	94.80	5.20	333	[22]
	Ga East and Cape Coast	20.10	18.10	46.50	15.30	O>A>B>AB	—	—	205	[21]
	Wa, Accra, Oti, and Tamale	18.31	23.48	55.09	3.12	O>B>A>AB	90.53	9.47	6094	[19]
	Total	18.67	23.20	50.26	7.87	O>B>A>AB	92.67	7.33	6332	
Observed frequency		19.65	21.74	54.72	3.89	O>B>A>AB	92.28	7.72	Rh+>Rh−	

### 3.5 Allelic Frequency Distribution of ABO and Rh(D) Blood Antigens in Ghana

The allelic frequencies of ABO and Rh blood antigens in Ghana were calculated using quadratic equation and the Hardy–Weinberg law of equilibrium of quantitative genetics. According to the equations, the allelic frequencies of A, B, O, Rh(D), and Rh(D) are denoted as I^A^(p), I^B^(q), i(r), I^D^(v), and I^d^(u), respectively. The allelic frequencies of A, B, and O were calculated to be 0.1227, 0.1376, and 0.7397 for I^A^(p), I^B^(q), and i(r), respectively. In addition, Rh(D)‐positive and Rh(D)‐negative allelic frequencies were 0.7222 and 0.2778 for I^D^(v) and I^d^(u), respectively. In this study, the observed phenotypic frequencies of A, B, AB, O, Rh(D) positive, and Rh(D) negative were 0.1965, 0.2174, 0.0389, 0.5472, 0.9228, and 0.0772, whereas the expected phenotypic frequency were 0.1966, 0.2225, 0.0338, 0.5472, 0.9228, and 0.0772, respectively. According to the chi‐square test, the goodness of fit for both ABO and Rh(D) blood antigens was not statistically significant (Tables 3 and 4).

**Table 3:** Allelic frequency distribution of ABO and Rh(D) blood antigens in Ghana.

Phenotype	Observed frequency	Genotypes	Expected frequency
A	0.1965	AA	p^2^ + 2pr = 0.1966
		AO/OA	
B	0.2174	BB	q^2 ^+ 2qr = 0.2225
		BO/OB	
O	0.5472	OO	*r* ^2^ = 0.5472
AB	0.0389	AB	2pq = 0.0338
Rh(D)	0.9228	DD/Dd	V^2^ = 0.5216
			V^2^ + 2uv = 0.9228
			2uv = 0.4013
Rh(D)	0.0772	dd	U^2^ = 0.0772

**Allele frequency of O**	
r^2^ = Phenotypic frequency of O	*Observed frequency of O = **0.5472**
r = √r^2^	
*r* = √0.5472	
***r*** = **0.7397**	
**Allele frequency of A**	
WA = AA + AO	*Observed frequency of A = **0.1965**
= P^2^ + 2pr	
= P^2^ + 2p (0.7397)	
= P^2^ + 1.4794p = 0.1965	
= P^2^ + 1.4794p − 0.1965 = 0	
According to the quadratic equation:	
$x=\frac{-b\pm \sqrt{b^{2}-4\mathrm{ac}}}{2a}$	
a = 1	
b = 1.4794	
c = −0.1965	
**p** = **0.1227**	
**Allele frequency of B**	
According to Hardy–Weinberg equation	*Observed frequency of B = **0.2174**
p + q + r = 1	
0.1227 + q + 0.7397 = 1	
q = 1 − 0.8627	
**q** = **0.1376**	
**Allele frequency of d**	
U^2^ = Frequency of d phenotype	*Observed frequency of d = **0.0772**
U^2^ = 0.0772	
U = √0.0772	
**U** = **0.2778**	
**Allele frequency of D**	
V = Frequency of D phenotype	*Observed frequency of D = **0.9228**
U + V = 1	
V = 1 − 0.2778	
**V** = **0.7222**	

**Table 4:** Comparison of observed and expected phenotypic frequencies of ABO and Rh(D) blood group system.

Existing allelic frequency					Existing genotypic frequency of ABO and Rh system						Expected genotypic frequency of ABO and Rh system					
A I_A_ = p	B I_B_ = q	O i = r	D ID = v	d Id = u	O	A	B	AB	Rh+	Rh−	O r^2^	A p^2^+ 2pr	B q^2^+ 2qr	AB 2pq	Rh+ V^2^+ 2uv	Rh‐U^2^
0.1227	0.1376	0.7397	0.7222	0.2778	0.5472	0.1965	0.2174	0.0389	0.9228	0.0772	0.5472	0.1966	0.2225	0.0338	0.9228	0.0772
Total = 1			Total = 1		Total = 1				Total = 1		Total = 1				Total = 1	

### 3.6 Comparison of ABO and Rh(D) Phenotypic Distribution in Ghana to Different Countries

The pattern of dominance of ABO in the current study, in Burkina Faso [42], Togo [43], Côte D'ivoire [44], Nigeria [9], Morocco [45], Algeria [46], India [3], the Philippines [47], and Iraq [2], was O>B>A>AB, whereas O>A>B>AB were reported in Ethiopia [1], Sudan [48], Mauritania [49], Somalia [50], Tanzania [51], Uganda [52], Saudi Arabia [53], and the United States [8]. Moreover, blood group A was predominant, and the blood groups occurred in the order of A>O>B>AB in Egypt [4], China [54], Croatia [55], and Turkey [56]. Interestingly, in Pakistan, blood antigen B predominates and blood antigens occur in the order B>O>A>AB [7]. Rh(D)‐positive was predominant in all countries, with Croatia (19.0%) [55], Turkey (14.98%) [56], and the United States (14.70%) [8] having the highest prevalence of Rh(D)‐negative blood groups. The lowest distribution of Rh(D)‐negative blood groups was found in China (1.02%) [54] and the Philippines (1.10%) [47] (Table 5).

**Table 5:** Comparison of ABO and Rh(D) phenotypic distribution in Ghana to different countries.

Countries	Phenotypic frequencies of ABO and Rh(D) blood antigens							
	A (%)	B (%)	O (%)	AB (%)	Comment	Rh+ (%)	Rh− (%)	Comment
Ghana (current study)	19.65	21.74	54.72	3.89	O>B>A>AB	92.28	7.28	Rh+> Rh−
Burkina Faso [42]	22.54	28.56	43.30	5.60	O>B>A>AB	92.24	7.76	Rh+> Rh−
Togo [43]	21.9	26.8	49.0	2.30	O>B>A>AB	92.63	7.63	Rh+> Rh−
Ethiopia [1]	28.41	21.24	44.65	5.71	O>A>B>AB	94.82	5.18	Rh+> Rh−
Nigeria [9]	21.0	28.0	47.0	5.0	O>B>A>AB	96.0	4.0	Rh+> Rh−
Sudan [48]	30.0	14.0	51.0	5.0	O>A>B>AB	97.4	2.6	Rh+> Rh−
Mauritania [49]	28.28	18.56	49.10	4.05	O>A>B>AB	94.23	5.77	Rh+> Rh−
Somalia [50]	26.50	11.27	60.30	1.93	O>A>B>AB	96.49	3.43	Rh+> Rh−
Tanzania [51]	24.4	19.1	52.4	4.0	O>A>B>AB	95.3	4.7	Rh+> Rh−
Uganda [52]	25.0	20.39	50.36	4.25	O>A>B>AB	97.97	2.03	Rh+> Rh−
Côte d'Ivoire [44]	22.51	23.53	49.74	4.40	O>B>A>AB	97.0	3.0	Rh+> Rh−
Egypt [4]	37.91	24.12	27.43	10.54	A>O>B>AB	91.42	8.58	Rh+> Rh−
Morocco [45]	32.86	15.80	46.80	4.53	O>B>A>AB	91.0	9.0	Rh+> Rh−
Algeria [46]	33.47	15.93	46.32	4.28	O>B>A>AB	—	—	—
China [54]	30.5	29.4	30.4	9.7	A>O>B>AB	98.98	1.02	Rh+> Rh−
India [3]	23.16	34.10	34.56	8.18	O>B>A>AB	94.13	5.87	Rh+> Rh−
Pakistan [7]	23.99	33.37	33.14	9.74	B>O>A>AB	90.63	9.37	Rh+> Rh−
Iran [5]	30.2	27.7	33.8	8.3	O>A>B>AB	88.2	11.8	Rh+> Rh−
Iraq [2]	24.30	26.02	38.80	10.86	O>B>A>AB	89.8	10.2	Rh+> Rh−
The Philippines [47]	24.0	24.9	45.5	5.7	O>B>A>AB	98.9	1.10	Rh+> Rh−
Croatia [55]	38.0	18.0	37.0	7.0	A>O>B>AB	81.0	19.0	Rh+> Rh−
Turkey [56]	41.88	15.26	34.92	7.93	A>O>B>AB	85.02	14.98	Rh+> Rh−
Saudi Arabia [53]	28.30	19.28	47.94	4.48	O>A>B>AB	92.06	7.94	Rh+> Rh−
The USA [8]	37.10	12.20	46.70	4.10	O>A>B>AB	85.3	14.70	Rh+> Rh−

## 4 Discussion

Ghana is home to a population of approximately 33,107,275 and accommodates people of diverse religions and cultures in its 16 regions [12]. Knowledge of the distribution of ABO and Rh(D) blood antigens is essential for clinical practice, especially for improving the safety of blood transfusion services. This systematic review determined the distribution of ABO and Rh(D) blood antigens and their corresponding allelic frequencies in Ghana. This review analyzed 134,227 records from 11 regions in Ghana from January 2000 to February 2024.

In this study, blood group O was predominantly observed among Ghanaians, and this prevails in other populations in Africa [1, 9, 42, 44, 46, 48, 50, 51] and Asia [2, 3, 47]. The inheritance and/or possession of ABO blood antigens in a population may be directed by genetic variabilities, environmental factors, and natural selection for population survival [8]. However, the possible relationship between ABO antigens and diseases, including malaria, has been recognized. Rowe et al. provided insights into the association between ABO and malaria and reported that individuals with blood group O may have significant protection against severe malaria [57]. A recent study in Northern part of Ghana found that individuals with blood group O were about seven times and thrice less likely to suffer severe malaria than their counterparts with blood antigens A and B, respectively [10]. The mechanism of the protective advantage of blood antigen O against severe malaria may not be clear, but has been associated with the limited rosetting of plasmodium‐infected red blood cells in individuals with blood group O, and this prevents the excessive sequestration of plasmodium‐infected erythrocytes in endothelial cells and eventually limits the occurrence of severe malaria [57]. Earlier, Haldane [58] had postulated in his “Malaria theory” that specific human genetic variant is protective against malaria in certain populations, and in malaria‐endemic areas in Africa, about 40%–80% of the population possess blood group O [59]. This may explain why blood group O is more prevalent in malaria‐endemic areas, but this would require further studies to determine the exact role blood antigens may play in malaria pathogenesis.

In this study, blood group B was the second most predominant blood group after O, compared to blood groups A and AB, but the frequencies of blood antigens A and B were variable in various regions of Ghana. While blood group A was second to O in Central and Western regions, blood antigen B occurred more frequently than blood antigen A in the Greater Accra, Volta, Ashanti, Northern, and North East regions. The highest frequency of blood group O was found in the Central region, followed by the Ashanti and Eastern regions, with the lowest O in the North East region of Ghana. However, the frequencies of blood antigens A and B were similar in the Eastern and Brong Ahafo regions. Greater Accra region, located on the Southern coast of the Gulf of Guinea, had the highest frequency of blood antigen B in Ghana, and this is similar to a previous study in which B was predominant among inhabitants residing around the coastal areas of the Ganges River in India [60]. Blood antigen B could confer protective mechanisms against severe Vibrio cholerae infection in cholera‐prone coastal areas [7, 60]. This may explain why blood group B was the highest in Greater Accra where more than half of Ghana's cholera cases from 1998 to 2017 have been reported [61].

The Rh(D)‐positive blood group was predominant among Ghanaians in this review, and this occurs globally. In this study, the prevalence of Rh(D)‐negative blood group in Ghana was 7.72%, which is similar to data from Burkina Faso [42], Togo [43], and Saudi Arabia [53]. However, the prevalence of Rh(D)‐negative observed in this study is higher than the prevalence reported in Ethiopia [1], Nigeria [9], Sudan [48], Mauritania [49], Somalia [50], Tanzania [51], Uganda [52], Côte d'Ivoire [44], China [54], India [3], and the Philippines [47]. Moreover, the prevalence of Rh(D)‐negative blood group reported in the United States [8], Turkey [56], Croatia [55], Iraq [2], Iran [5], Pakistan [7], and Morocco [45] was higher than that among the prevalence among Ghanaian population in this study. Whereas Croatians (19.0%) [55], Turkish (14.98%) [56], and Americans (14.70%) [8] had high prevalence of Rh(D)‐negative blood group, the lowest distribution of Rh(D)‐negative blood groups was reported among the Chinese (1.02%) [54] and the Filipinos (1.10%) [47]. The distribution of ABO and Rh(D) blood antigens could be influenced by potential factors, such as genetic drift, gene flow, genetic hitchhiking, geographical and environmental factors, natural selection for population survival, ethnicity, and race [7, 8], and this could account for the variations in the distribution of the blood antigens from one population to another. Individuals with Rh(D)‐negative blood groups in the present study were more common in the Northern region, followed by the Greater Accra, Volta, and Western regions, with the lowest frequency of Rh(D)‐negative individuals found in the North East region of Ghana. The imbalance in the distribution of ABO and Rh(D) blood antigens in distinct regions of Ghana may be related to the influence of environmental factors and natural selection for population survival.

In the current study, the standard assumption of the Hardy–Weinberg model was employed to estimate the allelic frequencies of blood antigens in Ghana. The allelic frequencies of A, B, O, Rh(D) positive, and Rh(D) negative were calculated as 0.1227, 0.1376, 0.7397, 0.7222, and 0.2778 for I^A^(p), I^B^(q), i(r), I^D^(v), and I^d^(u), respectively, which is consistent with a previous study [3] but contrary to the study by Rehman and Shi in Pakistan [7]. When the observed phenotypic frequencies of A (0.1965), B (0.2174), AB (0.0389), O (0.5472), Rh(D) positive (0.9228), and Rh(D) negative (0.0772) were compared to their respective expected phenotypic frequency of 0.1966, 0.2225, 0.0338, 0.5472, 0.9228, and 0.0772, there was no significant difference, which is comparable to earlier studies [3, 7].

### 4.1 Strengths and Limitations of the Study

This study elucidates the phenotypic distribution and allelic frequency of the ABO and Rh(D) blood groups in various regions of Ghana. However, this study had some limitations. This study could not cover the entire Ghanaian population since the distribution of ABO and Rh(D) blood groups had not been reported in five out of the 16 regions from 2000 to 2024, and some regions had fewer papers published with smaller sample sizes. Chances of population overlap could occur because multiple articles were reported from a particular region from 2000 to 2024. Lastly, this study included only D out of the five antigens (D, C, E, c, and e) of the Rh blood group system, but the expression of D antigen could be influenced by other Rh antigen variants.

## 5 Conclusion

Blood group O is the most common blood group, followed by group B, A, and AB in Ghana. Ghanaians with Rh(D) antigen constitute 92.28%, and 7.72% do not possess the Rh(D) antigen. Phenotypic frequencies of blood antigens A and B vary in different regions of Ghana. Knowledge about the frequency distribution of blood antigens in a particular area or whole country provides information for regional and national blood banks, population genetics, and forensic investigations. Future nationwide studies with larger sample sizes are recommended to assess the distribution of ABO, Rh, and other blood group systems to improve the quality of blood transfusion practices and predict the link between diseases and blood antigens.

## Author Contributions

**Charles Nkansah:** conceptualization, data curation, formal analysis, writing–original draft, writing–review and editing. **Felix Osei‐Boakye:** conceptualization, data curation, formal analysis, writing–original draft, writing–review and editing. **Samuel K. Appiah:** writing–original draft, writing–review and editing. **Gabriel Abbam:** writing–original draft, writing–review and editing. **Moses Banyeh:** formal analysis, writing–original draft, writing–review and editing. **Samira Daud:** writing–original draft, writing–review and editing. **Richard V. Duneeh:** writing–original draft, writing–review and editing. **Simon B. Bani:** writing–original draft, writing–review and editing. **Boniface N. Ukwah:** writing–original draft, writing–review and editing. **Charles A. Derigubah:** writing–original draft, writing–review and editing. **Victor U. Usanga:** writing–original draft, writing–review and editing. **Emmanuel Appiah‐Kubi:** writing–original draft, writing–review and editing. **Ejike F. Chukwurah:** writing–original draft, writing–review and editing.

## Conflicts of Interest

The authors declare no conflicts of interest.

## Supporting information

Supporting information.

## Data Availability

All data extracted and included in the study have been added as supporting files.
